# Predictors of Adverse TB Treatment Outcome among TB/HIV Patients Compared with Non-HIV Patients in the Greater Accra Regional Hospital from 2008 to 2016

**DOI:** 10.1155/2020/1097581

**Published:** 2020-08-04

**Authors:** Kenneth Mawuta Hayibor, Delia Akosua Bandoh, Adwoa Asante-Poku, Ernest Kenu

**Affiliations:** ^1^Noguchi Memorial Institute for Medical Research, University of Ghana, Ghana; ^2^School of Public Health, University of Ghana, Ghana

## Abstract

**Introduction:**

The convergence of TB and HIV dual epidemics is a major public health challenge in Ghana as well as many developing countries. Treatment outcome monitoring is a vital part of the surveillance needed to successfully eliminate TB. The impact of HIV status and demographic and treatment-related factors on adverse TB treatment outcome has not been studied in the Greater Accra Regional Hospital. This study determined factors associated with TB treatment outcome in patients with TB-HIV coinfection and TB-only infection in the hospital.

**Method:**

A cross-sectional study was carried out in the Greater Accra Regional Hospital. We reviewed TB treatment cards of patients who received treatment for tuberculosis in the hospital from 2008 to 2016. Data on treatment outcome and sociodemographic and clinical characteristics were extracted on TB-only-infected and TB/HIV-coinfected patients. The chi-squared test and binary and multiple logistic regression models were used to assess factors associated with adverse treatment outcome.

**Results:**

Out of the 758 patient records analyzed, 174 (22.9%) were TB-HIV-coinfected patients. Overall treatment success for all TB patients was 88.1% (668/758). About 11.9% (90/758) of the patients had an adverse treatment outcome, including treatment failure 0.9% (7/758), defaulting 0.9% (7/758), and death 10.0% (76/758). TB-HIV-coinfected patients' treatment success was 78.1% (136/174). TB-only patients' treatment success was 91.4% (532/582). Independent predictors of adverse treatment outcome were found to be as follows: being HIV positive (aOR: 3.85, 95% CI: 2.19-6.75; *p* < 0.01); aged 65 and above (aOR: 1.76, 95% CI: 1.44-1.54; *p* = 0.01); and previously failed TB treatment (aOR: 5.02, 95% CI: 2.09-28.87; *p* < 0.01).

**Conclusion:**

Treatment outcome for TB-HIV-coinfected patients is below the WHO target. HIV status, age, and category of patient of the TB patients were associated with adverse treatment outcome. Strengthening the TB/HIV collaborative efforts by stakeholders is required for good treatment outcomes.

## 1. Introduction

Tuberculosis (TB) and Human Immunodeficiency Virus (HIV) are diseases of global public health concern despite the existing prevention and control strategies [1]. TB is caused mainly by Mycobacterium tuberculosis, and it is curable. However, without early detection and diagnosis, it is difficult to treat. HIV is another infectious disease; however, there is no known cure at the moment. The HIV pandemic poses a great challenge to the control of the TB epidemic by changing the natural progression of latent TB to active TB and also impacting on the epidemiology and clinical outcomes of TB [2]. TB is a leading killer among people living with HIV. At least one in four deaths among people living with HIV can be attributed to TB, and many of these deaths occur in resource-limited settings especially in sub-Saharan Africa [2].

There were 10.4 million incident cases of TB worldwide in 2015, of which about 10% were coinfected with HIV and there were about 1.4 million deaths, of which 400,000 deaths were among people coinfected with HIV [3]. The sub-Saharan African region bears the highest global TB/HIV burden, and over 50% of TB cases are coinfected with HIV [3].

Ghana is one of the high-burdened TB/HIV countries in the world [4]. The prevalence of TB in Ghana in 2015 was estimated to be 356 per 100,000 population, and 22.5% of the TB patients are coinfected with HIV [5]. The impact of HIV on TB, and the implications for TB and HIV control, has been acknowledged as a public health challenge in Ghana.

There was a policy change in Ghana in July 2007 on collaboration of TB and HIV activities. The TB/HIV policy which was in line with the WHO recommendations consists of three linked sets of activities: effective implementation of the Stop TB Strategy for TB control, improved HIV prevention and care, and implementation of a TB and HIV coinfection clinic [2]. This policy was fully implemented in the Greater Accra Regional Hospital (GARH) in December 2007. The GARH has one of the best collaborative TB and HIV activities supported by the National Tuberculosis Control and National HIV/AIDS Control Programs with cross-referral mechanisms with other health facilities. However, since the integration of TB/HIV activities, there are few published reports on TB treatment outcomes among TB/HIV patients compared with non-HIV patients likewise the impact of HIV status and demographic and treatment-related factors on adverse TB treatment outcome in the GARH.

The World Health Organization (WHO) recommends that TB treatment outcomes be monitored, as monitoring is a vital part of the surveillance needed to successfully eliminate TB [6]. Treatment monitoring also serves as a tool to assess the TB treatment quality provided by the health care system [7]. There are six possible TB treatment outcomes: the patient may be cured, completed treatment, treatment failure, default treatment, transferred out to another facility, or died. Treatment success is when a patient is cured or completed treatment. Regardless of the number of reported TB cases in a setting, key treatment outcomes are expected to improve in line with WHO treatment outcome benchmarks. Currently, the international benchmark for assessing countries' performance with respect to cure rates is 85%.

This study reports on the prevalence of HIV and treatment outcome of TB patients who received treatment at a Directly Observed Therapy Short-course (DOTS) center in the GARH from 2008 to 2016. It further reports on the background and clinical characteristics associated with adverse treatment outcome among the TB patients. The GARH was one of the first health facilities in Ghana that integrated TB-HIV activities.

## 2. Methods

### 2.1. Study Design and Setting

This was a cross-sectional study of treatment outcomes of all TB cases registered from January 2008 to December 2016 in the GARH. The GARH serves the whole of the Greater Accra Region of Ghana, and it is a major referral center. The hospital has a DOTS center and a dedicated TB laboratory for TB cases' diagnosis, treatment, and monitoring according to NTP guidelines and protocols. All patients diagnosed with TB are made to undergo HIV counseling, testing, and treatment.

### 2.2. Data Collection and Study Variables

Data for this study was obtained from the TB register and patients' treatment cards at the DOT center of the hospital. The TB register is a standardized document used by all DOTS centers in Ghana. From March to May 2017, data was extracted from the TB register and treatment cards using a specially developed data extraction form. The background characteristics (age, sex, distance of residence to the DOTS center, and treatment supporter availability), clinical characteristics (category of patient, HIV status, diabetes status, TB diagnostic category, and adverse reaction to the anti-TB drug), and treatment outcome (cured, treatment complete, died, defaulted, and treatment failure) were the variables of interest. The WHO definitions of the six-treatment outcome category, type of patient, and TB diagnostic category were used [6].

### 2.3. Data Handling and Analysis

The abstraction forms were cross-checked for completion. Data were coded, entered, and cleaned using Microsoft Excel 2016 and imported into STATA 14 statistical software for analysis.

Data were summarized using frequencies and percentages to describe background characteristics, clinical characteristics, and treatment outcomes. A chi-squared test of association was used to determine significant differences in characteristics between TB-only and TB/HIV coinfection patients. Bivariate and multiple logistic regression models were used to identify significant predictors of adverse TB treatment outcomes in all TB patients. Statistical significance was set at a *p* value of 0.05 at 95% confidence level.

### 2.4. Ethical Approval

Ethical approval was obtained from the Ethical Review Committee of Ghana Health Service, Research and Development Division, Accra (Reference No. GHS/RDD/ERC/Admin/App/17/434). Permission to conduct the study was obtained from the Central Administration and the Deputy Director of Nursing Services of the DOT center at the GARH. To ensure privacy and confidentiality, data abstraction was done in a room provided by the hospital authority. Access to the data obtained from the TB cards was limited to the study investigators. No identifying information such as names was captured from the records. Data collected were solely used for research purpose. Confidentiality and anonymity were maintained from data entry into the data abstraction forms and into the computer before data analysis.

## 3. Results

A total of 761 TB patients were registered at the Greater Accra Regional Hospital from January 2008 to December 2016. Treatment outcome was not documented for three patients; therefore, only 758 cases were included in the analysis.

### 3.1. Background Characteristics of TB Patients by HIV Status

Table 1 describes the background characteristics of TB cases in the GARH, 2008-2016. Majority of the patients were in the age group 25-44. There were 526 (69.4%) males, and there were 232 (47.1%) females. With the patients' area of residence, 77% of TB/HIV-coinfected patients and TB-only patients resided in an urban area. Most of the patients live less than 5 km away from the hospital. Almost all the patients in both categories had treatment supporters. There was a statistically significant difference in age (*p* < 0.001) and sex (*p* < 0.001) between TB-HIV patients and TB-only patients. However, there was no evidence of statistical difference between the two categories of patients with respect to distance to the hospital (*p* = 0.51), area of residence (*p* = 0.97), and available treatment supporter (*p* = 0.13).

### 3.2. Disease Characteristics of TB Patients with or without HIV

Majority of the patients in both categories were mostly newly diagnosed cases. Seventy-nine percent of the TB/HIV patients were new cases likewise 73.5% for the TB-only patients. More than half of the TB-only patients were smear positive (57.7%). Most of patients' (both TB/HIV and TB-only) chest X-rays were suggestive of TB. Sixty-two percent of the patients that experienced adverse drug reactions were TB/HIV-coinfected patients.

There was a statistically significant difference in TB classification (*p* < 0.01) between TB-HIV-coinfected patients and TB-only patients. There was no significant association between TB-HIV-coinfected patients and TB-only patients with type of patient (*p* = 0.25), chest X-ray (*p* = 0.09), duration of treatment (*p* = 0.75), diabetes (*p* = 0.27), and adverse treatment reaction (*p* = 0.052) (Table 2).

### 3.3. TB Cases by HIV Status

**Figure 1:**
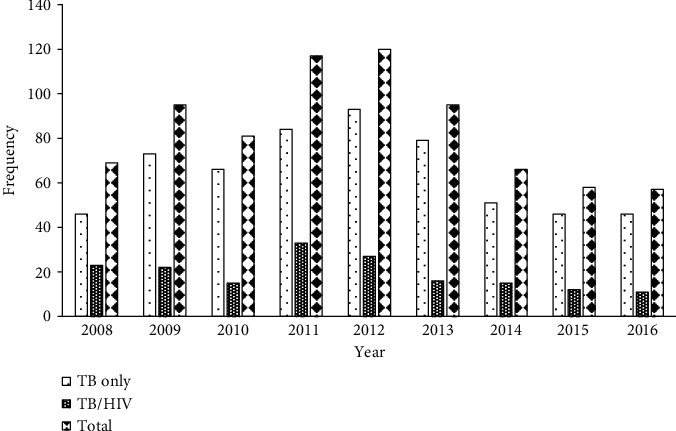
TB cases by HIV status registered in the Greater Accra Regional Hospital, 2008-2016.

The highest number of TB cases over the period was 120, which was recorded in 2012, and the lowest was in 57 in 2016 (Figure 1). The proportion of TB patients coinfected with HIV over the period was 22.9% (174/758). There was a gradual increase in the number of TB cases from 2008 to 2012 except 2010, and then there was a gradual decrease till 2016, and this was likewise for TB-HIV-coinfected cases.

### 3.4. Treatment Outcomes of TB Patients with or without HIV

Treatment success for patients coinfected with TB and HIV was 77.0% (134/174) (95% CI: 0.70-0.83). TB-only patients' treatment success was 91.4% (534/584) (95% CI: 0.89-0.94). The overall treatment success was 88.1% (668/758) (95% CI: 0.86-0.90) Twelve percent (90/758) of the patients had an adverse outcome. Thirty-five (20.1%) of the HIV-positive patients and 43 (7.4%) of the HIV negative patients died (Table 3).

### 3.5. Predictors of Adverse TB Treatment Outcomes

Multiple logistic regression revealed that after adjusting for six other variables, HIV status, age, and category of patient were significantly associated with adverse treatment outcome (Table 4). TB patients coinfected with HIV had 4.5 times higher chance of adverse treatment outcome compared to non-HIV TB patients (aOR: 4.5, 95% CI: 2.6-7.8; *p* < 0.01). Likewise, the odds of experiencing adverse treatment is 4 times as great in patients' aged above 64 years than that of patients below 15 years (aOR: 4.0, 95% CI: 1.44-1.54; *p* = 0.01). Patients who have previously defaulted treatment had 8.1 times higher odds of having adverse treatment outcome compared to new patients (aOR: 8.12 95% CI: 2.19-30.08; *p* = 0.002). The odds of adverse treatment outcomes was 5.2 times higher among patients that previously failed TB treatment compared to new TB patients, though this was not statistically significant (aOR: 5.2, 95%: CI 0.49-55.36; *p* = 0.17).

## 4. Discussion

This study was set to determine factors associated with TB treatment outcome in patients with TB-HIV coinfection and TB-only infection in the GARH. The main findings of the study were as follows: 22.9% of all TB patient records reviewed were coinfected with HIV; the overall treatment success for TB patients was 88.1% while that of the patients coinfected with HIV was 77.3%. Adverse treatment outcome was associated with HIV coinfection, previously defaulted treatment, previously failed treatment, and aged above 64 years.

Studies conducted in the African region on TB/HIV coinfection showed varied values ranging between 2.9% and 72.3%, with pooled prevalence of 23.5% [8]. The HIV prevalence recorded in this study is comparable to the Ghana national average of 22% [9] and a study in Ethiopia (25%) [10]. However, it is higher than the current global estimate of 15% and much lower than the African regional estimate of 36% [5]. This finding reveals why Ghana is considered to be one of the high-burdened TB/HIV countries in the world by the WHO [4].

WHO recommends treatment outcome monitoring among TB patients as an essential component for TB control and surveillance. The overall treatment success recorded in this study is higher than most of the studies conducted in the African subregion [11–14]. The high overall treatment success recorded in this study may be attributed to prudent following of the WHO treatment guidelines. An implication of the high treatment success rate recorded is that Ghana is likely to meet the End TB targets if this trend continues.

TB/HIV-coinfected patients in this study had more adverse treatment outcomes than patients with TB only. Globally, TB-associated mortality in coinfected patients is three times higher than mortality among TB-only patients [5]. TB/HIV-coinfected patients in this study had more adverse treatment outcomes than patients with TB only. Moreover, coinfected patients have an increased risk of experiencing adverse treatment outcomes compared to TB-only-infected patients. Adverse treatment outcomes associated with TB/HIV coinfection have been shown by several studies [8, 11, 15–18]. The reasons for the adverse treatment outcome may be due to immunosuppression [19], drug interactions between rifampicin a major anti-TB drug and some antiretroviral agents [20, 21], suboptimal drug concentrations of anti-TB drugs [22], and a malabsorption of the anti-TB drugs [23]. Also, late diagnosis of HIV [24], alternation of the clinical manifestation of TB [22, 25], lack of a rapid and sensitive TB diagnostic test [26, 27], and unavailability/inaccessibility of antiretroviral therapy [28] may be the cause of the adverse treatment outcomes.

Globally, TB/HIV coinfection rates have been falling since 2008 [5]; however, in our study, the rate increased gradually and peaked at the year 2011, before a gradual decrease year after year (Figure 1). This gradual decrease in our study suggests that the current TB and HIV collaborative activities in the hospital must be encouraged to further reduce the coinfection rate. Moreover, a study in five districts in the Volta Region of Ghana revealed a stable TB/HIV coinfection from 2012 to 2015 [11]. TB-associated mortality in coinfected patients globally is three times higher than mortality among TB-only patients [5]. TB/HIV-coinfected patients in this study had more adverse treatment outcomes than patients with TB only. Coinfected patients have an increased risk of experiencing adverse treatment outcomes compared to TB-only-infected patients.

Our findings revealed that males were more susceptible than females, and this is consistent with a study done in Southern Ethiopia [12], but this is in contrast with studies conducted in the Volta Region of Ghana [11], Gondar [13] and Gambella [14]. Our findings might be due to most males spending more time at their workplaces and outside their homes than females, which predisposed them for susceptibility of TB infection. However, another possible explanation could be that females underutilized the DOTS services at the hospital.

Majority of the TB patients were aged between 25 and 45. Persons within this age group are usually sexually active. HIV/TB coinfection is more common in the sexually active age group (18). This finding is consistent with several studies that have also identified TB/HIV coinfection rates to be higher in persons within these age groups [9, 19, 20]. However, it is contrasted by a study where majority of the coinfected patients were below 15 years [11].

This study observed that patients who previously failed treatment and patients who return after previously defaulting treatment have an increased risk of developing adverse treatment outcomes compared to newly registered patients. This shows that more attention needs to be given to this category of patients and the reason for their previous treatment failure needs to be investigated.

It was also observed that TB patients aged above 64 were more likely to experience adverse treatment outcome compared to patients' aged below 15 years. This finding is consistent with studies conducted in Gondar University Teaching Hospital, Northwest Ethiopia, where the elderly were more likely to die than the younger ones [13]. Patients aged 64 and above may have adverse treatment outcome due to their immune systems as they age.

This study is a cross-sectional study and based only on records that were available in the GARH. It should be seen that the study incorporated data of patients with completed information of their treatment outcome at the hospital. We could not collect additional data needed, which could affect treatment outcome such as educational status and occupation, to confirm or refute our findings. In particular, we did not have access to ART history among our group of HIV-coinfected patients, and therefore, we could not assess its effect on our findings. It is likely though that most of them were not receiving ART and care or were diagnosed late in the course of their HIV disease. Despite this limitation, the study provided useful information on the treatment outcomes of TB/HIV-coinfected patients and TB-only-infected patients in the GARH. Findings may be useful to the National TB Control Program, the National AIDS Control Program, and the hospital authorities.

## 5. Conclusion

Our findings indicate that TB-HIV coinfection among the TB patient records reviewed was very high. Treatment success for TB patients was 88.1% while that of the patients coinfected with HIV was 77%. Significant predictors of adverse treatment outcomes were TB/HIV coinfection, aged 64 years and above, and having previously failed treatment. Factors associated with adverse treatment outcomes in TB/HIV-coinfected patients and patients that have previously failed treatment should be investigated. Strengthening the TB/HIV collaborative efforts is required for good treatment outcomes.

## Figures and Tables

**Table 1:** Background characteristics of TB cases according to HIV status in the Greater Accra Regional Hospital, 2008-2016 (*n* = 758).

Characteristics	TB-HIV *n* (%)	TB-only *n* (%)	Total *n* (%)	*p* value
Age				<0.001^∗^
<15	27 (15.2)	37 (6.3)	64 (8.4)	
15-24	10 (5.8)	81 (13.9)	91 (12.0)	
25-34	36 (20.7)	134 (23.0)	170 (22.4)	
35-44	57 (32.8)	119 (20.4)	176 (23.2)	
45-54	36 (20.7)	93 (15.9)	129 (17.0)	
55-64	6 (3.5)	65 (11.1)	71 (9.4)	
>64	2 (1.2)	55 (9.4)	57 (7.5)	
Sex				<0.001^∗^
Male	92 (52.9)	434 (74.3)	526 (69.4)	
Female	82 (47.1)	150 (25.7)	232 (30.6)	
Area of residence				0.972
Urban	134 (77.0)	449 (76.9)	583 (76.9)	
Periurban	40 (23.0)	135 (23.1)	175 (23.1)	
Distance to the hospital				0.513
<5 km	133 (76.4)	460 (78.8)	593 (78.2)	
≥5 km	41 (23.6)	124 (21.2)	165 (21.8)	
Treatment supporter available^a^				0.212
Yes	173 (99.4)	570 (97.6)	743 (98.0)	
No	1 (6.7)	14 (2.4)	15 (2.0)	

^∗^Significant at *p* < 0.05. ^a^Significance was determined using Fisher's exact test.

**Table 2:** Disease characteristics of TB cases according to HIV status in the Greater Accra Regional Hospital, 2008-2016.

Characteristics (758)	TB-HIV *n* (%)	TB-only *n* (%)	Total *n* (%)	*p* value
Category of patient^a^				0.149
New	139 (78.9)	429 (73.5)	568 (74.93)	
Relapse	5 (2.9)	13 (2.2)	18 (2.37)	
Defaulter	0	12 (2.1)	12 (1.6)	
Failure	1 (0.6)	3 (0.5)	4 (0.5)	
Undefined	28 (16.1)	125 (21.4)	153 (20.2)	
Transferred in	1 (0.6)	2 (0.3)	3 (0.4)	
TB classification				<0.001^∗^
Smear positive	74 (42.5)	337 (57.7)	411 (54.2)	
Smear negative	65 (37.4)	109 (18.7)	174 (23.0)	
Extrapulmonary	35 (20.1)	138 (23.6)	173 (22.8)	
Chest X-ray				0.085
Suggestive	114 (65.5)	340 (58.2)	454 (59.9)	
Not suggestive	60 (34.5)	244 (41.8)	304 (40.1)	
Duration of treatment				0.750
<6 months	15 (8.6)	55 (9.4)	70 (9.2)	
≥6 months	159 (91.4)	529 (90.6)	688 (90.8)	
Diabetes				0.274
Yes	8 (4.6)	17 (2.9)	25 (3.3)	
No	166 (95.4)	567 (97.1)	733 (96.7)	
Adverse treatment reaction^a^				0.052
Yes	4 (2.3)	3 (0.5)	7 (0.9)	
No	170 (97.7)	581 (99.5)	751 (99.1)	

^∗^Significant at *p* < 0.05. ^a^Significance was determined using Fisher's exact test.

**Table 3:** Treatment outcomes of TB cases according to HIV status in the Greater Accra Regional Hospital, 2008-2016.

Variable	TB-HIV	TB-only	Total (%)	*p* value
Treatment outcome				<0.001
Cured	58 (33.8)	288 (49.3)	346 (45.6)	
Treatment completed	76 (43.7)	246 (42.1)	322 (42.5)	
Treatment failure	2 (1.2)	5 (0.9)	7 (0.9)	
Died	34 (19.5)	42 (7.2)	76 (10.0)	
Defaulted	4 (2.3)	3 (0.5)	7 (0.9)	
Overall treatment outcome				<0.001
Treatment success^∗^	134 (77.0)	534 (91.4)	668 (88.1)	
Adverse treatment^∗∗^	40 (23.0)	50 (8.6)	90 (11.9)	

^∗^Sum of treatment outcomes cured and treatment completed. ^∗∗^Sum of treatment outcomes treatment default, died, and defaulted.

**Table 4:** Logistic regression analysis of predictor variables of adverse treatment outcome among TB patients in the Greater Accra Regional Hospital, 2008-2016.

Variables	Treatment outcome		aOR	95% CI	*p* value
	Trt. success^∗^ *N* (%)	Adverse trt.^∗∗^ *N* (%)			
Age					
<15	52 (7.8)	12 (13.33)	Ref		
15-24	85 (12.7)	6 (6.7)	0.61	0.20-1.87	0.39
25-34	150 (22.5)	20 (22.2)	0.92	0.39-2.17	0.85
35-44	157 (23.5)	19 (21.1)	0.75	0.32-1.77	0.51
45-54	119 (17.8)	10 (11.1)	0.51	0.19-1.32	0.16
55-64	64 (9.58)	7 (7.8)	0.96	0.29-2.48	0.95
>64	41 (6.1)	16 (17.8)	4.00	1.54-10.34	0.004
Sex					
Male	469 (70.2)	57 (63.3)	Ref		
Female	199 (29.8)	33 (36.7)	0.94	0.57-1.597	0.81
Category of patient					
New	510 (76.4)	58 (64.4)	Ref		
Relapse	16 (2.4)	2 (2.2)	1.40	0.29-6.74	0.67
Defaulter	8 (1.20)	4 (4.4)	8.12	2.19-30.08	0.002
Undefined	128 (19.2)	25 (27.8)	1.33	0.45-3.89	0.60
Failure	3 (0.5)	1 (1.1)	5.22	0.49-55.36	0.17
Transferred in	3 (0.45)	0	1.00	—	—
TB classification					
Positive	376 (56.3)	35 (38.9)	Ref		
Negative	147 (22.0)	27 (30.0)	1.34	0.74-2.46	0.34
Extrapulmonary	145 (21.7)	28 (31.1)	1.73	0.59-5.08	0.31
Adverse treatment reaction					
Yes	4 (0.6)	3 (3.3)	3.90	0.78-19.48	0.10
No	664 (99.4)	87 (96.7)	Ref		
TB patient coinfected with HIV					
No	534 (79.9)	50 (55.6)	Ref		
Yes	134 (20.1)	40 (44.4)	4.48	2.59-7.77	<0.01

Trt. = treatment. ^∗^Sum of treatment outcomes cured and treatment completed. ^∗∗^Sum of treatment outcomes treatment default, died, and defaulted.

## Data Availability

The data used to support the findings of this study are included within the article.
